# Targeted cancer therapy: the initial high concentration may slow down the selection for resistance

**DOI:** 10.18632/aging.206046

**Published:** 2024-07-28

**Authors:** Mikhail V. Blagosklonny

**Affiliations:** 1 Roswell Park Comprehensive Cancer Center, Buffalo, NY 14263, USA

**Keywords:** lung cancer, resistance, brain metastases, METex14, capmatinib, rapamycin

## Abstract

Unfortunately, any targeted therapy is, always, started with low levels of the drug in the organism, selecting for drug resistance. One should propose that initial drug levels must be maximized, and durations may be minimized, ideally, as portions of preemptive combination of targeted drugs.

To ensure rapid selection of drug resistance in the cell culture, we first treat cancer cells with low drug concentrations, increasing drug concentrations over time. I hope no further explanation is needed.

In patients, treatment with targeted drugs is also mistakenly designed to expedite the selection of resistance, a fact that may shock my readers. Initially, cancer cells are exposed to low drug concentrations, which are then increased. This is achieved simply by administering the same daily dose to patients from day 1. For example, Cabozantinib is taken once daily. Cabozantinib has a long terminal plasma half-life (~120 hours) and accumulates 5-fold by day 15 with daily dosing.

I reiterate: the concentration increases 5-fold by day 15 due to its long half-life and consistent daily dosing of one tablet.

Low initial concentrations can be avoided by taking 5 tablets on the first day and then continuing with 1 tablet every week, for example. Another hypothetical regimen is 3 tablets for every 3 days and then discontinuing it. Levels of Cabozantinib remain high for the next one to two weeks (post-treatment remained activity).

Consider my case: I have multiple brain metastases driven by METex14, effectively targeted by capmatinib, the most selective and effective MET inhibitor. Capmatinib selects for resistant secondary mutations in the METex14 that can be targeted by Cabozantinib, although it is less effective and selective than capmatinib. It is possible that one of the metastases contains at least one capmatinib-resistant cell with secondary MET mutations, which could eventually make the metastasis resistant to capmatinib.

In my case, I propose using 5 tablets of Cabozantinib for 1 day every two months. Only 1 day every 2 months.

The mutant cell is exposed to relevant concentrations of Cabozantinib for one to two weeks (post-treatment remained activity).

Another example. Afatinib (EGFR/HER2-4 inhibitor) is used to treat EGFR-mutant-dependent lung cancer (it is not my case, as mine is METex14-dependent). Afatinib has a half-life of 37 hours. Steady-state is achieved within 8 days of once-daily dosing, with overall accumulation ratios of 2.0–2.7 for C max and 2.5–3.4 for AUC. Instead, we should achieve Steady-state on day 1. In some cases, the first day levels should be higher than ever further and the course of treatment may be brief.

In my case, the most common off-target mechanism of resistance involves overexpression of EGFR and HER2-4. I prefer very high dose treatment with afatinib for just 1 days in combination with capmatinib. After stopping afatinib, its levels remain high for several additional days (post-treatment remained activity), and capmatinib must continue to be used. Afatinib alone is ineffective in my case (METex14), but afatinib targets potential resistance to capmatinib. (Note: Without capmatinib, METex14-positive cells would not be killed by the off-target resistant drug. A combination of two drugs must be used, including high levels of afatinib post-treatment).

Another example: trametinib (an MEK inhibitor) is usually given 1 tablet (2 mg) every day from day 1. The estimated elimination half-life is 3.9 to 4.8 days. Trametinib accumulates with daily repeat dosing with the accumulation ratio of 6.0. Steady state was achieved by day 15. This is reminiscent of Cabozantinib.

One may suggest that steady-state-like levels (or even higher levels) may be required for the very beginning of treatment. Especially, in brief applications use. For drugs with long half-life, the drug may be given only on day 1 because high levels of the drug remain for a long time in the organism, no matter what. These are post-treatment levels and I call this the reversal level curve (Figure 1). I suggest to use reversal schedules in targeted drug combinations (Figure 2). I’ve depicted the schema I have used for me for very specific reasons that are beyond the topic of this editorial (My battle with cancer: exceptional chapters from part II). It is worth mentioning that inhibition of MEK and mTOR should be used together, because rapamycin may activate MEK, and Trametinib may activate the mTOR pathway.

**Figure 1:**
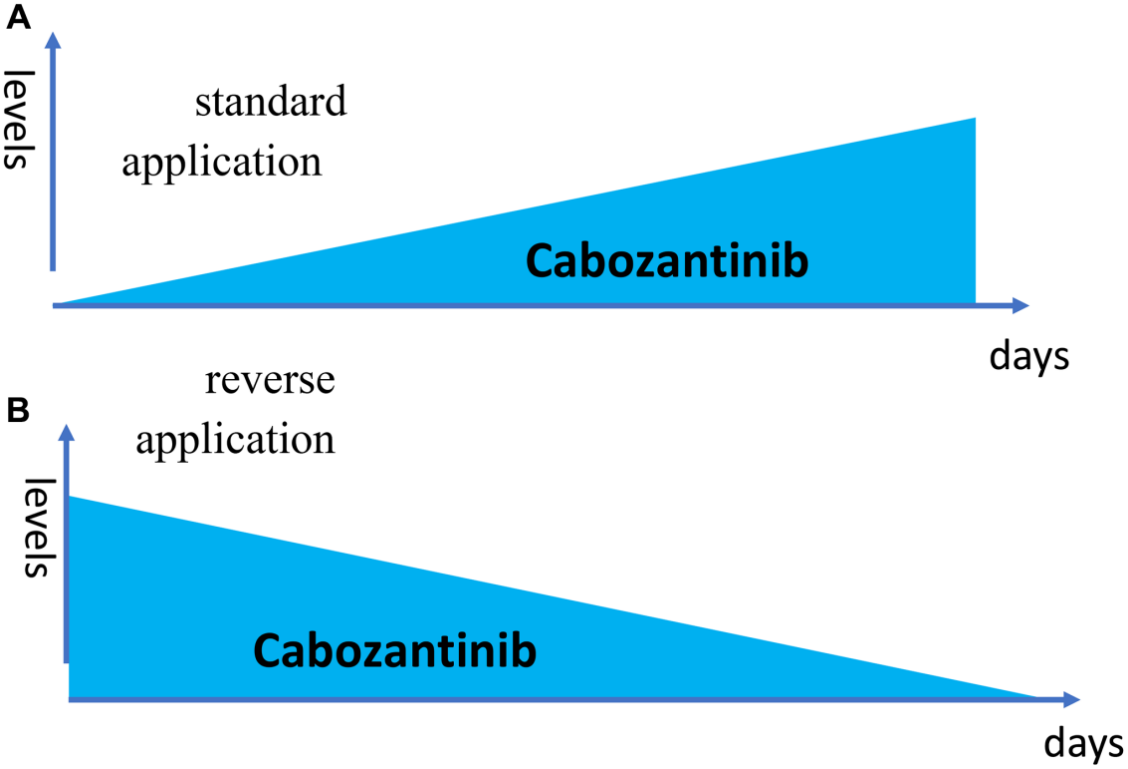
**Reverse dose application.** (**A**) The drug is given every day 1 tablets. (**B**) The drug is given on day 1 only 6 tablets (just one as an example).

**Figure 2:**
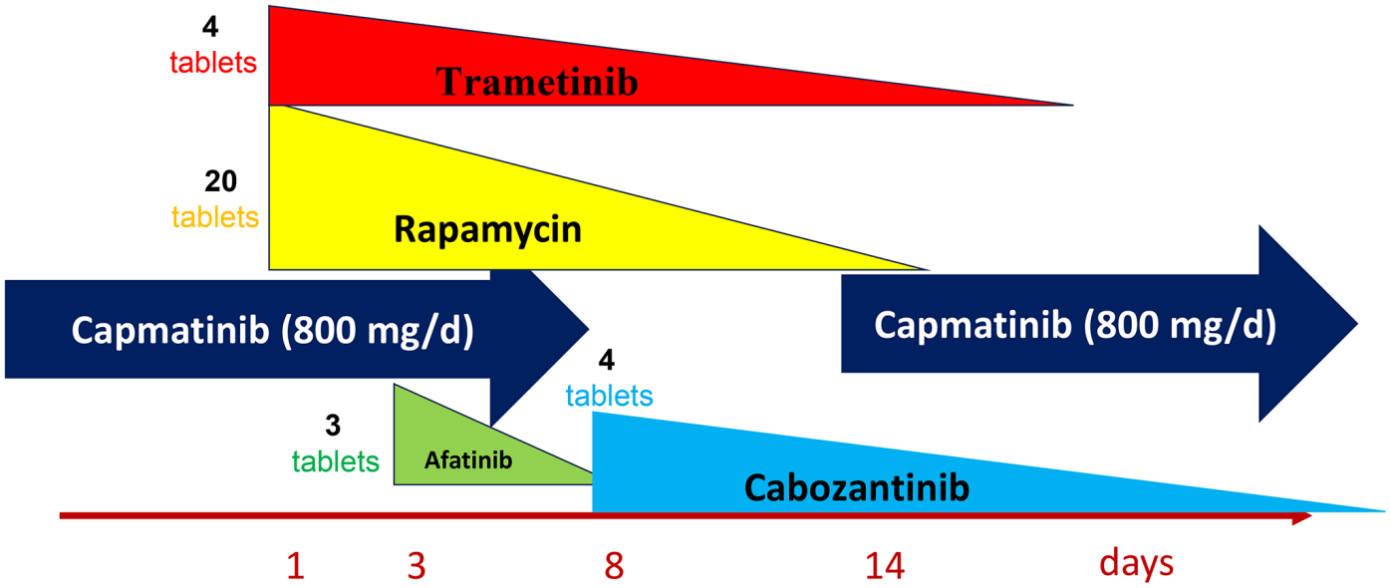
**One of my targeted combinations.** Capmatinib – standard everyday use. All other medications - reverse dose application -are given on day 1 only. The number of tablets on day1 are shown.

In conclusion, to decelerate the acquisition of drug resistance, targeted therapy must immediately target the tumor at a higher than steady-state drug concentration. The courses may be brief, if the doses are high, but that is a topic for another story. Importantly, as previously discussed, targeted drugs should be used in appropriate combinations [1, 2].

Starting treatment with a higher dose is common in medicine. For instance, starting with high doses is typical for antibacterial antibiotics to avoid resistance. Often, antibiotics are used at double (or higher) doses on the first day, as a high single dose, or even intravenously.

Also, rapamycin is given at a loading dose on the first day, which is three times higher than the maintenance dose in organ transplant patients. For another example, dexamethasone is often started with a load.

**Appendix**

**“My battle with cancer. Part 1.” Oncoscience. 2024 Jan 3; 11: 1-14.**

**Abstract:**

In January 2023, diagnosed with numerous metastases of lung cancer in my brain, I felt that I must accomplish a mission. If everything happens for a reason, my cancer, in particular, I must find out how metastatic cancer can be treated with curative intent. This is my mission now, and the reason I was ever born. In January 2023, I understood the meaning of life, of my life. I was born to write this article. In this article, I argue that monotherapy with targeted drugs, even when used in sequence, cannot cure metastatic cancer. However, preemptive combinations of targeted drugs may, in theory, cure incurable cancer. https://www.ncbi.nlm.nih.gov/pmc/articles/PMC10765422/

**Forthcoming “My battle with cancer: exceptional chapters from part II.”**

**Abstract:**

For a divine reason, I was destined for cancer with multiple brain metastases: to create the book “My Battle with Cancer,” a far-reaching endeavor for which I was born. Surprisingly to others, I experience moments—no, entire days—of happiness and joy. My journey through cancer, as both a patient and a researcher, has gifted me insights into cancer cures, born from a mind that remains active, often even in sleep. Unfortunately, one thing happened on May 20, 2024, hurting me, but this is not a part of this book. Nevertheless, I am finishing the book with the hope of increasing the lifespan to the normal duration for future incurable cancer patients. Among other topics, I propose preemptive combinations of targeted drugs to prevent resistance to the key cancer driver and thus sustain long-lasting remission; classification of targeted combinations; intermittent targeting; reverse dose applications; increasing targeting of brain metastases; and the harmful effects of standard WBRT, Avastin, and immunotherapy-induced hyper-progression.
